# Social Determinants of Health and Linkage to HIV Care Among Groups at High Risk for Not Linking to HIV Care

**DOI:** 10.21203/rs.3.rs-6534966/v1

**Published:** 2025-05-16

**Authors:** Lauryn Mills, Estefania Reyes Sepulveda, Zoe Miller, Jasmine Jones, Gabriela Hernandez, Sophia M. Ly, Jules Canfield, Tim Flanigan, Paul Goulet, Raynald Joseph, Emily Hurstak, Jennifer A. Palmer, Martha Sanchez, Kaku So-Armah, Amanda M. Fitzpatrick

**Affiliations:** Brown University; Boston Medical Center; Boston Medical Center; Boston Medical Center; Boston Medical Center; Boston Medical Center; Boston Medical Center; Brown University; Brown University; AIDS Care Ocean State; Boston Medical Center; Center for Health Optimization and Implementation Research (CHOIR), VA Boston Healthcare System, Boston University Chobanian & Avedesian School of Medicine; Brown University; Boston University; Boston Medical Center

**Keywords:** Social determinants of health (SDoH), Human immunodeficiency virus (HIV), People living with HIV (PLWH)

## Abstract

**Background:**

1 in 5 people with new HIV diagnoses are not linked to medical care after diagnosis. Persons who are Black/African American, Hispanic/Latinx, young, and previously or currently use drugs via injection have low rates of linking to HIV care. We explored perceptions of patients living with HIV about how social determinants of health (SDoH) affect linkage to care.

**Methods:**

We recruited patients from a large safety-net hospital in Boston belonging to one of the groups with high risk for low linkage to HIV care. We conducted semi-structured interviews in-person or via Zoom/telephone about how the five domains of SDoH (i.e., health care access and quality, education access and quality, economic stability, neighborhood and built environment, and social and community context) impacted their HIV care experience. We conducted rapid thematic analysis of data by creating: 1) transcript summaries organized by each SDoH and 2) a matrix comprising the transcript summary data.

**Results:**

Participants were 50% female, 72% Black/African American, 22% Hispanic or Latina/o/x, and 56% with current or past use of drugs via injection. 89% had experienced at least one gap in HIV care since diagnosis. Themes related to how healthcare access and quality impacted linking individuals to HIV care included enrollment in HIV drug assistance programs and rapport with (or stigma from) healthcare providers including mental healthcare providers. Themes related to education access included explicit education on HIV treatment from providers and adapting HIV education to patient’s needs (e.g., tailoring to patient’s self-efficacy for independently finding credible health information). Economic stability themes included access to affordable food, and reliable transportation. Themes on social and community context included emotional support (or stigma) from family, friends, and/or partners.

**Conclusions:**

Participants perceived multiple social needs as impacting HIV care linkage. Their most salient perceptions related to positive and negative roles of family/friend support, self-perception as a person with HIV, and HIV stigma. Addressing SDoH may improve linkage to HIV care in disproportionately affected populations.

## Introduction

In 2021, 1 in 5 of the 33,606 people in the US with newly diagnosed HIV were not linked to life-saving HIV care within 1 month of diagnosis; well below the *Ending the HIV Epidemic* target of 95% linkage.^1,2^ Linkage to HIV care is the act of making an initial appointment with an HIV medical provider within 30 days of being diagnosed with HIV. It is a crucial early step in HIV treatment, a necessary precursor to taking antiretroviral therapy (ART), and critical to improving rates of viral suppression.^3,4^ This gap in linkage to care is not evenly distributed in the U.S. population. People who use drugs via injection, Black/African American people, people ages 13–24, and transgender women experience lower than average rates of successful linkage to HIV care.^1^ Though linkage to care by those of Hispanic/Latin(a/o/x) ethnicity is slightly above average, disparities in HIV incidence and prevalence persist: people of Hispanic/Latin(a/o/x) ethnicity comprise about 18.5% of the population but make up 29% of the population of people living with HIV.^5^

Social Determinants of Health (SDoH) refer to the conditions in which individuals live, work, grow, and age and the structures and systems in which people operate, which, while not necessarily medical, influence our health.^6,7^ SDoH include economic and social status, education, food and job security, working conditions, social inclusion and discrimination, structural conflict, and access to healthcare.^7^

There are several ways social factors may impact health including for people with HIV. Individuals exposed to intrapersonal, interpersonal, and/or institutionalized discrimination based on race, ethnicity, language, gender, and/or sexual preference are at risk for reduced physical, emotional and financial support, increased behaviors like unhealthy substance use, and increased chronic disease in comparison to those without such exposures.^8–10^ Institutional racism resulting in high rates of mass incarceration, poverty, and sexual violence for African Americans contributes to increased risk for HIV exposure and barriers in access to healthcare, and linkage to HIV care.^11^ Immigration policies leading to family separation and deportation decrease Latino trust in state and federal government and increase the potential for racial bias in healthcare settings.^12,13^ The effects of this may drive reduced receptiveness to public health advisements on HIV testing and prevention, which may be compounded by historic mistreatment of people of color in addressing HIV.^11^

Prior studies suggest SDoH influence multiple aspects of the HIV care cascade from diagnosis to long term viral suppression.^14–16^ These studies suggested social and economic disadvantage are prevalent among people living with HIV (PLWH), are cumulatively associated with HIV care outcomes, and disproportionately impact people of color.^15,16^ However, these studies were limited as they did not qualitatively explore how and why SDoH impact linkage to HIV care to identify potential intervention opportunities.^12^ The few qualitative studies did not focus on groups with lower linkage to care to enable tailoring of future interventions where need may be greatest.^16^

Our study explores how SDoH impacts linkage to HIV care among members of these groups who are at the highest risk for not linking to HIV care. For this study, linkage to care includes patient entry into HIV care after diagnosis or after a significant gap in care. We also explored clinical retention through discussing patients’ current HIV care maintenance. We examined the relationship between HIV care linkage and five domains of social determinants of health: health care access and quality, education access and quality, economic stability, neighborhood and built environment, and social and community context.

## Methods

### Sample

We recruited people with HIV from a large tertiary safety-net hospital’s adult Infectious Diseases practice in Boston, Massachusetts from July – September 2023. Eligibility criteria included individuals ages 18–30, Black/African American, Hispanic and Latin(a/o/x), and having current or past injection drug use. We enriched our sample with individuals who have experienced a gap in care. We defined a gap in care as the following: patient is (1) newly diagnosed with HIV and had or is scheduled for an intake appointment with a nurse, but has not engaged with the provider within 30 days of HIV diagnosis; (2) transferring HIV care (i.e., out of state) and had or is scheduled for an intake appointment with a healthcare provider, but has not yet engaged with the provider; or (3) dropped out of HIV care (i.e., 12+ months or 2+ missed appointments within 12 months) and had or is scheduled for an intake appointment with a healthcare provider, but has not yet reengaged with the provider.

### Data collection

One-on-one interviews (45–60 minutes in duration) were conducted by members of the Providence/Boston Center for AIDS Research (CFAR) Learning Community (CLC) Team using a semi-structured interview guide designed with input from qualitative researchers and investigators working in HIV clinical care. CLC is a longitudinal undergraduate summer research pathway program funded by the National Institute of Allergy and Infectious Disease CFAR Diversity, Equity and Inclusion Program Initiative (CDEIPI).^17,18^ We used the Healthy People 2030 SDoH Framework in the creation of the interview guide to identify five domains of exposure: Healthcare Access and Quality, Education Access and Quality, Economic Stability, Neighborhood and Built Environment, and Social and Community Context. Healthcare access and quality is the access to comprehensive, high-quality health care services and includes things such as having health insurance, affording health care services and medications, and having a primary care provider. Education access and quality are educational opportunities such as graduating from high school and college. Economic stability is earning a steady income that allows an individual to meet their needs (healthy foods, health care, and housing). Neighborhood and built environment include how a neighborhood impacts an individual’s health and wellbeing, with the goal of creating neighborhoods that promote health and safety. Social and community context is people’s relationships and interactions with family, friends, co-workers, and community members, and how these interactions impact health and well-being. We asked each participant to reflect on how each domain of social determinants of health influenced their ability to link with and engage in HIV medical care. We further probed how these experiences could have been improved from the participants’ viewpoint. All participants were compensated for their time with a ClinCard valued at $50.

### Ethical approval

This study was reviewed and approved by the Boston University Institutional Review Board (H-43727), in accordance with The Belmont Report: Ethical Principles and Guidelines for the Protection of Human Subjects of Research. Those eligible provided written informed consent prior to participation in study activities.

### Analysis

We conducted deductive qualitative thematic analysis of semi-structured interview data using rapid thematic assessment.^19,20^ Rapid approaches to analyzing qualitative data have been found to accelerate discovery timelines and allow for actionable intervention development while maintaining scientific rigor. The three stages of analysis include: (1) summarizing transcripts in a template organized by questions from the semi-structured interview guide (performed by E.R., L.M., G.H.), (2) transferring the templated summary data to a pilot-tested matrix to conceptualize patterns within and across participants’ narratives (E.R.), and (3) identifying the thematic structure based upon the deductively emergent patterns (E.R.). Several steps were taken to ensure analytic rigor. First, a doctoral-level qualitative methodologist and health services researcher trained the three analysts in rapid assessment procedures. Second, thematic analysis continued until no new themes emerged (saturation). Third, discordant themes or disagreement in theme assignment were adjudicated through group discussion.

## Results

### Demographic characteristics of interview participants and major themes

We interviewed 18 eligible participants. Black/African American participants represented 72% of our sample, Hispanic/Latin(a/o/x) represented 22%, and the remaining 6% identified as white (Table 1). Fifty-six percent mentioned having a history of substance use. 50% were 55 years or older, 39% were between 31–54, and the remaining 11% were aged 18–30 years. Half the sample self-identified as female.

Across the social determinants of health, participants emphasized the following themes: 1) a negative experience during HIV diagnosis impedes linkage to care while a positive experience and subsequent connection to a trusted primary care provider facilitates linkage to care, 2) ignorance of HIV and treatment contributes to fear and inaccurate perceptions of life with HIV, 3) competing responsibilities and lack of transportation inhibits people with HIV from attending HIV care appointments, 4) support from family and support groups reduce stigma and encourage adherence to HIV treatment.

### Impact of Healthcare Access and Quality on Linkage to HIV Care

Interview participants shared their experience with health care access and quality that influenced their ability to initiate or sustain linkage to HIV medical care (Table 2). We define healthcare access and quality as the ability of individuals to readily obtain necessary healthcare services including preventive care, diagnosis, and treatment in a timely manner while also receiving care that is effective, safe, patient-centered, free of stigma and bias, and delivered with appropriate standards. Negative experiences, such as feeling judged, can deter patients from following up with their care; further exacerbating the delays in starting their HIV treatment. Many participants described the day they were diagnosed and remembered the year, the environment (clinic, hospital, penitentiary, etc.), and their interaction with the healthcare staff. Participants described how they felt once they received their diagnosis, despite the amount of time that has passed, with one sharing: *“I remember the day. Everything stopped. In my eyes, nothing moved, like my world just suddenly ended.”* Another participant described some of the emotions they felt the day of their diagnosis: *“Confused, devastated, depressed, mad, really upset.”*

A theme that emerged among older study participants (ages 55+) was reluctance to initiate or engage in HIV treatment due to participants’ knowledge of and/or personal experience with older medications. Specifically, many mentioned Zidovudine (AZT), the first antiviral drug used to treat HIV infection, being a barrier for care: *“I didn’t take the medicine. I didn’t take the AZT…because that was killing people back then.”* Though AZT is not currently used to treat HIV, participants in the age category of 55+ revealed it has lasting impacts on their desire to engage in HIV treatment. Many participants relayed that they decided not to take AZT because of its adverse side effects that participants experienced personally, witnessed, or learned of from others who took the medication.

When describing the environment where they were diagnosed and how their diagnosis was given by the provider, participants had varying experiences. Some participants felt uncomfortable when given their diagnosis and felt discriminated against. *“Back then, everybody was afraid. Everybody will wear gloves and suits and cover their nose and everything because nobody knew what was going on…it was a lot of discrimination.”* Another participant felt similarly, “*they were kind of wearing a shield when they were drawing blood.”* One mentioned that being diagnosed in a penitentiary influenced how providers gave their diagnosis: *“They called you back and they said, you tested positive. You can go now. Just like that. Yeah, I guess you’re in jail, you’re supposed to be punished. So, here goes another punishment.”*

On the other hand, some participants described positive experiences when receiving their diagnosis. A few participants attributed this to their team of health care professionals that had positive attitudes and made them feel comfortable. A participant acknowledged having a positive experience with the nurses: *“The nurses are all nice people…”* Similarly, a participant mentioned that they trusted their team of physicians: *“[I] have a great team of doctors from the start to the end.”* One participant even mentioned: *“I can tell him [my doctor] anything.”*

In discussing the experiences participants had with their HIV care team, two participants mentioned that their physician encouraged her to continue her HIV treatment:

1. He [Doctor] explained it to me like this. He said, imagine a brick wall. Every day you don’t take it, take a brick out the wall… And eventually, if you don’t take it, the wall is going to fall down.
2. He [Doctor] was very nice. Very kind…He told me that I have to be careful and sometimes if you don’t take your medication, sometimes things will happen to you, you will get sick more and sometimes you can die, but you can live long as you take care of yourself.

A common theme of having positive relationships with HIV care providers and nurses revealed an overall positive outcome on participants feeling encouraged to continue seeking HIV treatment and communicating with their provider about their HIV needs. A positive experience with providers that are compassionate with HIV diagnosis and education along with having positive encouragement from an HIV care provider allowed participants to have an open communication style with their provider and voice their concerns when needed.

Most participants mentioned having health insurance, specifically MassHealth, and that it facilitated their ability to get their HIV medication. Some participants mentioned using the support of drug assistance programs: “*I have HDAP, HIV Drug Assistance Program…which is good.”* On the other hand, several participants mentioned that the ability to afford their medication is difficult: *“I really can’t afford the insurance and then I really can’t afford the medication either. But I do what I can to get it. That’s why I’m here today.”* The ability to afford medication becomes increasingly difficult for participants that are getting treated for more than one disease. One participant mentioned finding it difficult to supply their medication for this reason: *“I have to pay for my other medications too. I got high blood pressure and stuff like that. But the HIV medication is the most expensive.”*

Participants communicated how HIV impacted their mental health in the early stages of their diagnosis and would have liked their HIV care providers to link them to mental health care. Many participants reported that they struggled significantly with their mental health after their diagnosis. Participants mentioned vividly remembering the day of their diagnosis and associating feelings of trauma, devastation, depression, and suicidality. Many communicated that they felt sad and upset following the weeks after their diagnosis. One participant suggested that mental health support should be required instead of recommended for people recently diagnosed with HIV:

I was offered a mental health specialist, I feel like it should have been more instead of a recommendation…like…you have to do it, because more than once in that beginning time [time of diagnosis] I thought about taking my own life multiple times, because I’m like, I’m not going to live… I’m not going to be able to see the future or have kids or have a husband or anything like that. That’s the main thing that goes through your mind. And especially going into adulthood is like, now you feel like everything is gone. You don’t have anything.

Many others mentioned that they had used the support of counselors, psychiatrists, and other physicians to help them process their diagnosis and support their mental health.

### Impact of Education Access and Quality on Linkage to HIV Care

Participants were asked about their experiences learning about HIV and how well their provider explained the disease during diagnosis (Table 3). A participant who is deeply committed to the dissemination of knowledge around HIV and community building, mentioned the importance of education:

Ignorance is fear. If you are ignorant of something, you will fear it. That’s why education

is important. To educate somebody, that’s the best thing that you can do. And I think this

hospital could do just a little bit more with the education and pushing nurses to go a little more the extra mile with certain information.

Several participants mentioned that some of the information they learned had not been from physicians at all. Some discussed having prior knowledge about HIV from outside sources: *“All you heard is what you heard from the street.”* After learning more about HIV from their HIV care provider, a participant said, *“I felt like it was a death sentence, but I’ve learned so much about it now and I know it’s not a death sentence.”*

Participants were asked if they had done any outside research (i.e., internet searches, articles, books) to learn more if there was anything they did not understand from their physicians. One participant said, *“I found out through reading…The first thing I read was how to deal with AIDS and I continued from there.”* Another participant had mentioned that learning about HIV on their own was very helpful: *“I’m always doing research. I’m the type of person to go online and check.”*

### Impact of Economic Stability, Neighborhood and Built Environment on Linkage to HIV Care: Facilitators and Barriers

We asked participants how their economic stability, neighborhood and built environment affected their ability to access HIV care (Table 4, Table 5). A participant explained that there has never been a time they did not have enough to eat because the robust resources in Boston supplemented them with the things they needed during their experience of homelessness: *“I’ve been homeless before. But you can eat in Boston… you just gotta know where to go. You never starve here.”* Another participant described expressing food insecurity to a provider and being connected with the hospital food bank to access healthy options: *“Because I go to the food bank here. They give you a lot of vegetables. A lot.”*

Additionally, participants were asked how other competing responsibilities (such as being a caregiver to a child or family member) contributed to their ability to go to their HIV appointments on time. One participant described how this additional responsibility sometimes, but not always, prevents them from getting to their HIV appointments.

My mother, she is disabled. She’s been disabled for most of my life. I don’t live with her, my brother does, but occasionally, she does need me to come, but it makes it easy most of the time, because she’ll have an appointment at the same time [as my HIV-related appointments]. But if not, then it’s like we’re both trying to figure out how to do X, Y and Z.

When asked how transportation impacts their ability to get to HIV care appointments, participants described walking, using public transportation, relying on family, or, for a handful, driving to appointments. One participant who relied on walking described how weather could impede their travel:

I would walk from my house to [hospital]. It wasn’t too difficult because it was summer, thank the Lord. So when it would be winter and I wouldn’t walk, it would be a little hard, because just where the location [of the hospital] is with Methadone Mile [an area in Boston where neighborhood services including methadone clinics provide care to a large number of people who are experiencing homelessness and substance use disorders].

The cost of public transportation came up as an issue for participants with and without consistent housing. One participant said that while transportation was consistent, “*economically it is a problem. So, I got to get some assistance… through [hospital]. Sometimes they help me out with my [transportation pass].”* Several participants identified the hospital resources as a facilitator in transportation to appointments and easing the financial burden: “*I do drive but I don’t have my own car but like I said, I was so sick so I got rid of my car because I couldn’t afford to do all this stuff and that. But I do have a guy from [hospital] there for me…”*

Those who relied on family tended to have another mode of transport as well, either walking, public transport, or services from the hospital. One expressed concern of relying too much on family for transportation to appointments: “*I usually [have] my father or mother but that’s what I’m working on now – trying to get my own vehicle, just to not be such a burden*.”

### Social and Community Context on Linkage to HIV Care: Facilitators and Barriers

When speaking on how their social lives changed after their diagnosis, every participant noted fear of stigma, wariness of sexual intimacy, discrimination, social isolation, and/or self-isolation (Table 6). Most participants in the older age range experienced fear of HIV stigma and discrimination by both friends and doctors:

Back then, I used to keep it to myself. Just to keep it to myself, I won’t tell anybody, I won’t tell my family, my friends or anybody, just kept it to myself. That way nobody was discriminating against me…. The nurses and doctors back then, they kind of discriminated against me. Because like I said, they didn’t know anything about the disease.

Some participant mentioned that they didn’t feel comfortable discussing their diagnosis with anyone: *“Truthfully, [I] didn’t talk to anyone about it until five years after my diagnosis… It was so traumatic that I just wanted to put it behind and not think about it at all. Especially being 18, 19 about to start college.”*

Participants of all ages expressed hesitation to tell anyone around them. Those who did share with their family or roommates – only a handful of participants – reported growing closer to their family and roommates as a result: “*When I feel frustrated, I call my friends… I’ve grown closer to them. I’ll give the example of my roommate. She helps me remember to take my medication – when I talk about my sisters, she’s my sister.”*

However, even in these cases, the community available to these participants was often limited to a small circle. One participant who had shared their diagnosis only with their mother, who recently passed, said, “*Every time I think about it, it reminds me I am going to die and my mom would say ‘no, as long as you do what [the doctors] said’… she was my whole support system.*” A handful of participants noted how knowing another PLWH made handling their diagnosis and care easier: “*At first it was kind of hard because I didn’t expect it. But, you know, my brother also is HIV [*+*] … He’s been HIV [*+*] ... since 1998 or something. But he’s been taking care of himself very well and taking his medication. So we always keep [up with] each other, you know, maintaining ourselves.”*

Several participants’ statements show how society’s perception of HIV has changed over time, or since their diagnosis. Education came up repeatedly as a facilitator for reducing stigma of the disease. Where one participant, at the time of their diagnosis, “*thought it was a death sentence,*” another participant reported, “*You kind of get educated about it. I knew it wasn’t a death sentence because I had a family member that has it too, and she’s had it maybe like 20, 30 years and she’s just fine.”* Another participant described how public perception of HIV has changed since their diagnosis:

Doctors and nurses were even afraid to treat us, because they thought that by touching us, by treating us, we were going to pass the virus to them. So it was really very difficult. So there are so many trainings and seminars that were introduced by the government, because the government got funding from the US from all over the world. So they put in a lot of money to educate everybody and especially the healthcare workers. And then slowly people started to accept us.

While education and disclosing their diagnosis with roommates and family has decreased some stigma and feelings of isolation for participants, several participants reported closing themselves off to romantic or sexual intimacy, citing fears of passing their diagnosis on to a loved one – “*I’m always scared of giving it to someone else.”* Others feared rejection due to stigma/inability to be loved by someone else due to their diagnosis: “*I’m going to be alone for my whole life. I’m never going to have someone who will love me and want to be with me, because I was told and especially around the stigma of it all makes it harder for you to be like, oh yeah, I’m going to…have somebody, they love me, they care about me, so on so forth*.”

One participant spoke about the feeling of belonging they experienced when their partner accepted them after they shared their diagnosis, stating: “*My significant other, we just got together three years ago. And I told him in the very beginning… And he said that’s fine. He knows multiple people who have it, it doesn’t bother him… So it felt good to feel accepted for what I had and all of that*.”

Another participant spoke to the feeling of belonging and active participation in a circle of peers via a support group, and how it changed the trajectory of their life:

I was introduced to a support group and when I went there, I was welcomed with loving arms from other people who looked like me, who shared the same status. So I started going for those support group meetings, and they were teaching us about positive living, coping with your status…a lot of education… I moved from being that corpse to being now a human being. And my life started picking up, and I started going for seminars, for trainings and then I started teaching. I started doing consultancy work in my country and out of my [home] country and my life just changed. But it was a very long process.

### Participant’s Perceived Needs to Improve Linkage to HIV Care

When asked what helps and hinders them in accessing HIV care, as well as their suggestions for improvement, participants identified HIV education, welcoming medical environments, care coordination and case management, support groups, therapy, and affordable medication as facilitators in improving HIV care, including going to appointments and taking medication (Table 7). Participants stressed the need for education specifically around medication, especially for individuals experiencing other risk factors:

We need to get educated, not just say, here’s your medication and that’s it. Some people won’t even take it. The people using drugs and drinking, they’re not gonna take the HIV medication. You know what I mean? So, we need to educate these people more so they can understand this. Yeah, if you don’t take your medication, you’re gonna die. So people gotta be more aware about the disease.

Another participant noted how with advancements in medicine, it can be difficult to know what medication is best to take – “*Every day a different medicine comes up, but…They don’t explain to the people how, which medicine is the best you can take because this or because that. You know it’s coming up like that, but we don’t know if it’s good or not.*”

Several participants described their appointment environments as hostile spaces; “*The way we are treated when we go in that door over there. Oh, the first thing all we want is drugs and that’s not it. I think it should be less hostile. A less hostile environment to really get people to come in*.” Stigma towards people with HIV who also use drugs contributes to the care gap experienced by several participants and can deter people from seeking treatment or disclosing drug use history to healthcare providers. Related to the concept of caring about the individual outside of their diagnosis, another participant mentioned hospitals staff’s involvement with helping patients gain access to non-medical necessities: “*I tried to talk to the nurses in family health (about non-medical case management like housing or food assistance), so I will have an appointment next week.*” One participant directly referenced AIDS Action, a Boston-based organization providing HIV-related services in New England aiding with non-medical necessities like those mentioned above: “*I’ve been to AIDS Action. All kinds of things that you can learn, how to paint… they give you tickets to movies to the theater; they have lunch, dinner, and an exercise room. They have clothing if you need it. They have help if you need help paying your rent or something. They have meals that they give to you.*”

Having a community of PLWH that share common experiences allowed participants to connect and combat isolation. One participant noted that although these environments are helpful, it is important to be intentional and establish groups where members have commonalities besides their HIV diagnosis. They recommended establishing support groups of individuals close in age, as experiences and lived experience can differ based on age of diagnosis, evolution of HIV knowledge and education, and current life circumstances. One participant also argued for more nuanced groupings to increase comfort in sharing and sense of belonging.

I feel like not just support groups, but support groups for different ages, because I’ve been invited to multiple support groups, but these support groups have like children in them. I’m an adult, so it feels kind of weird to be at a support group with young children. Yeah. But they’re experiencing this just like we are, but we’re both experiencing it differently.

In the same vein, another participant said that therapy would help: “*Maybe sometimes I feel like talking with somebody. I should get therapy maybe one time or two times a month to help me out.*” Support groups have provided a safe space for these participants to share experiences, reduce stigma, improve coping mechanisms, enhance medication adherence and overall improve their quality of life.

In terms of access and ability to take medication, several participants talked about the financial barrier to medication and appointments due to both the medication cost itself as well as insurance.

*Just cheaper medicine. I mean, I would want the best medicine, but I don’t know how to pay $50 for one bottle. That’s about it*.” Another said “*So when it comes to health insurance and everything and having so much issues, getting medication or not having insurance or having insurance, it makes it harder to want to even go to the appointment…*

## Discussion

Our interviews with PLWH illustrated how SDoH impacted experiences of linking to HIV care. Given that the cohort of participants chosen represents those with lower rates of linkage care, their perspectives provide valuable insights to the factors that have enabled or prevented their access to HIV care. Themes related to how healthcare access and quality impacted linking individuals to HIV care included enrollment in HIV drug assistance programs and rapport with (or stigma from) healthcare providers including mental healthcare providers. Themes related to education access included explicit education on HIV treatment from providers and adapting HIV education to patient’s needs (e.g., tailoring to patient’s self-efficacy for independently finding credible health information). Economic stability themes included access to affordable food, and reliable transportation. Themes on social and community context included emotional support (or stigma) from family, friends, and/or partners. Participants did not directly address how issues like housing and experiences of homelessness impacted their ability to link to HIV care. However, housing is an important factor in physical and mental health; affordable, safe, and stable housing allows for more resources available to pay for health care, limits financial stressors, and has positive impacts on wellbeing.^21^

These themes overlap with themes from other studies investigating the impact of SDOH on HIV treatment and care of marginalized populations. A qualitative review of studies focusing on Hispanic/Latina women with HIV found barrier themes identified in our study including HIV-related stigma from health professionals, as well as additional barriers including legal consequences of seeking HIV services and language barriers.^22^ A qualitative review on the social determinants of HIV treatment among Black older women in the Southeastern U.S. found facilitators identified in our study including supportive social networks and HIV-related knowledge, as well as barriers including low income, health insurance, substance use, and stigma.^23^ These findings support our qualitative study results and illustrate the determinants of linkage to HIV treatment that may be influenced by a person’s identity. Our work extends these existing data by including the perspectives of several vulnerable groups and asking for patient-centered suggestions for improvement.

Our participants suggest the following strategies for improving linkage to HIV care:

### 1) Create a welcoming and inclusive environment for all patients.

HIV is a stigmatized health condition^23^ and the experience when receiving HIV diagnosis may influence one’s likelihood to link to care. Research highlights how perceived discrimination from health care workers and lack of guidance and follow-up are barriers to linkage to HIV care.^24^ Thus, creating a welcoming environment is an important part of providing care to patients. One patient recollected a time where HIV care took place in the basement of a hospital and felt that the patients were “hidden.” Now, HIV care takes place on the top floor where natural light and a view of the city allows patients to feel recognized and comfortable. Further, many participants in our study are from marginalized communities and have other stigmatized, co-occurring conditions. An inclusive environment that recognizes the identities of patients may allow for patients to feel more comfortable when seeking care. For example, a participant with a co-occurring substance use disorder felt that they were labeled as “drug-seeking”, and this negatively influenced their care experience. It is important that physicians providing HIV care recognize and mitigate the effects of for co-occurring conditions like substance use disorders.

### 2) Emphasizing mental health care and support groups for recently diagnosed PLWH.

People with HIV are at a higher risk for mental health disorders and depression is one of the most common mental health conditions faced by PLWH.^25^ Given that participants mentioned that they experienced a severe decline in their mental health and that support groups were helpful forms of coping with their diagnosis and a meaningful way to find connection, providers should include and emphasize the importance of seeking mental health support and offer resources for support groups as part of patient’s care plan throughout their course of treatment, starting at the time of diagnosis and/or first follow-up. The time of diagnosis has been described as scary and overwhelming by participants. Participants may not be ready for support groups at this time; however, continually offering resources throughout their care (especially to patients who have transferred their place of care) allows individuals to seek mental health resources when they are ready to initiate. Further, support groups can and should be personalized to the identity of the patients in order to increase comfort in disclosing personal experiences and a sense of belonging. This can be accomplished through support groups for PLWH with shared backgrounds (e.g., race/ethnicity, age, and gender identity/sexual orientation).

### 3) Creating education and support for PLWH to initiate and continue to take prescribed medication(s).^26^

Many participants described experiencing difficulty, hesitancy, and/or reluctance with initiating/continuing their HIV treatment because of barriers surrounding medication. Barriers described included: 1) cost of medication(s), 2) difficulty in remembering or wanting to take medication, and 3) negative experiences with past medications. Many participants described multiple programs that reduce or eliminate the cost of HIV medication.^27^ However, some participants experienced lapses in coverage. Providers may work with patients to ensure that patients are taking part in the programs they are eligible for, assist them in any application processes, and check in to ensure they do not lapse in coverage. Additionally, some participants described that reminders to take medications from friends and/or family encouraged them to take their medications and continue to engage in treatment. However, many participants lack this kind of social support and had trouble in remembering or wanting to take their medication(s) daily. A study has found that sending simple text messages to PLWH significantly improved ART adherence in their study population.^28^ Medical providers and case workers may develop a reminder system for patients who wish to enroll and would benefit from this daily encouragement.

In addition, older participants acknowledged reluctance and distrust with HIV medication because of the adverse effects of AZT. When these participants were diagnosed, AZT was the only HIV medication approved by the FDA during the late 80s to 90s.^29^ The reluctance to initiate or continue HIV treatment among this demographic of patients may be due to the lack of options for HIV medication during that time and adverse effects. Due to these past experiences, it is important for physicians to specifically acknowledge this when encouraging their patients that are 55+ to begin or continue their HIV treatment plan. Compassionate patient education and tailoring their conversation of HIV treatment plans could be particularly beneficial to this group.

### Study Limitations and Future Directions

Our study has several limitations worth noting. First, although the CDC identified individuals 13–24 as the age group with the lowest HIV care linkage, our study recruited from an adult clinic that limited participation to individuals 18+. Second, transgender women were identified by the CDC as the group with the greatest percentage dropping out of care after diagnosis, yet no transgender women were recruited for this study. Further investigation should include recruiting from clinical setting serving young adults as well as clinics with a higher representation of transgender women. Additionally, this study was conducted in a single urban setting. Facilitators and barriers may differ between urban and rural settings, warranting further investigation on facilitators and barriers to HIV care linkage among low-linkage groups in rural settings. Nevertheless, we believe our study offers fruitful perspectives and recommendations for improving linkage to HIV care among multiple marginalized populations.

## Conclusion

We assessed how the SDoH impact access and linkage to HIV care, and summarized recommendations that patients believe will improve the healthcare experiences of PLWH. This can inform efforts to improve linkage to HIV care, and consequently the rest of the HIV care cascade, among PLWH from minoritized and marginalized populations.

## Figures and Tables

**Table 1.** Demographics of participating individuals

Demographics	Interview No. (N = 18)	%
**Race/Ethnicity**		
Black or African American	13	72.2
Hispanic/Latina/o/x or Spanish Origin	4	22.2
White	1	5.6
**Sex/Gender Identity**		
Male	9	50
Female	9	50
**Age Range**		
18–30	2	11.1
31–54	7	38.9
55+	9	50
**History of Substance Use via Injection**		
Yes	10	55.5
No	8	44.5
**Gap in Care**		
Yes	16	88.9
No	2	11.1

**Table 2.** Impact of Healthcare Access and Quality on Linkage to HIV Care.

Healthcare Access and Quality	
HIV Diagnosis Experience	*“I remember the day. Everything stopped. In my eyes, nothing moved, like my world just suddenly ended.”* *“At first, it was scary, because nobody knew about the disease. People were just dying within weeks. The way they were dying was awful. And I just refused to take medication”*
HIV Diagnosis Environment	*“Back then, everybody was afraid. Everybody will wear gloves and suits, and cover their nose and everything because nobody knew what was going on. Yeah, it was a lot of discrimination.”* *“The letter came back saying that you need to come in. So I went in and the woman, she didn’t take me by myself, she just did everything on the floor.”* *“When I was tested before, I was tested without my consent, I was sick, very, very sick. I had nonstop diarrhea, and I became very, is it emaciate, what is that word? If you saw me, I looked like a walking corpse.”*
Healthcare Before & After HIV Diagnosis	*“I am a chronic asthmatic and an asthmatic since the day I was born. So I've always had a low immune system… So me going to the hospital would be once a year for a physical or something like that or if I was having an asthma attack, I would be taken to emergency.”* *“You know the story about back home, we don’t even go to the hospital. Why do we really go to the hospital? I only go to the hospital when I'm very, very sick. That’s when I go to the hospital. But other than that, back home people don’t go to the hospital, never. You only go there maybe when you have pain that is not going away, because we are so used to buying over the counter.” (016)*
Experience with Doctor(s)	*“They welcomed me very good, I had family in Shapiro building…I have a friendship relationship if that makes sense.”* *“He was very nice. Very kind. Even though I was crying at the moment, but he was very nice…He tell me that I have to be careful and sometimes if you don’t take your medication, sometimes things will happen to you, you will get sick more and sometimes you can die, but you can live long as long as you take care of yourself.”*
Experience with Health Insurance	*“Well, I mean, the insurance pays for most of that stuff.”* *“I don’t have to pay so my insurance cover for everything good.”* *“My employer provides insurance.”*
Experience with Pharmacy & Getting Medications	*“They will deliver the package [of medicine] to my house.”* *“With the coverage I have now and the coverage I had before, it’s still pretty hard to get medication.”* *“I mean, I would want the best medicine, but I don’t know how to pay $50 for one bottle. That’s about it.”* *“Actually, it’s $70, because I have to pay for my other medications too. I got high blood pressure and stuff like that. But the HIV medication is the most expensive.”* *“I didn’t want Benzoyl because I heard it eats your bones and I want to be normal.”*
Frequency of Doctor Visits and/or Multiple Health Conditions	*“I have a pacemaker…because it doesn’t beat normally or something… Recently, I've had cancer three times. I've had it cut out on the right side, I had it cut out on the left side and it’s back again on the left side.”* *“it seemed like every three weeks at first. And now one time, the doctor told me, okay you can come and see me in the next 90. I'm like, huh, that long and then, because me, myself, I've had it all. I had lymphoma. I caught the shingles. Oh, I was just trying to get over that cancer.”*
Impact of HIV on Emotions and Mental Health	*“It was like a taboo, like you don’t want to, you want to keep it private and you don’t want -- one of those things especially those days. You don’t want people to know because then life…with humans can be really bad.”* *“I learned that…what do you call it? When you're thinking about that… you feel like you're depressed, the depression feeling is more than the sickness. So I don’t put my mind in that, I don’t think about it.”* *“I was given to talk to, they had like, counselors and just general people that was there that can help that, people with the new diagnosis and stuff, and that I can just open up to. And they were really helpful and they still have been helpful at the hospital as well.”*

**Table 3.** Impact of Education Access and Quality on Linkage to HIV Care.

Education Access and Quality	
Knowledge & Understanding of HIV	*“Yeah, I'm always doing research. I'm the type of person to, like, go online and check...and see what this is and what can I do about this.”* *“Ignorance is fear. If you are ignorant of something, you will fear it. That’s why education is important. To educate somebody, that’s the best thing that you can do. And I think this hospital could do just a little bit more with the education and pushing nurses to go a little more the extra mile with certain information.”* *“Basically, I did my own research. It wasn’t anything that could really tell me I didn’t know about.”*
What Providers Could Have Done Differently to Explain HIV Diagnosis	*“To make a miracle to not have it. No, to be honest they did it, I wanted the answer to be different.”* *“The nurses are educated, I have good nurses now. They look at me like I'm not sick. They always like, 'Oh, you look so good.' So that makes me feel good.”* *“I was never requested for my consent, because consent is very important.* *Back home they do things differently. But they were doing it to save my life. So they tested me and they shared my results with the third party...And that subjected me to a lot of stigma and discrimination, and that is what even took me a long time to come to terms with my status. So there's that consent, and then that sharing information with other people and then people started ridiculing me, calling me a dead corpse, calling me all kinds of names, people telling me I was going to die and things like those. So it was very, very difficult for me. But I know here before they test you, this country they have to get your consent, And it’s very confidential when they test you, nobody gets to know your status. Even your very close people don’t get to know your status unless you tell it to them. And then here medication is available. So you get to choose. It’s not like those years back when it was only AZT. So it was either you take it or not. And the prices were also very prohibitive.”*

**Table 4.** Impact of Economic Stability on Linkage to HIV Care.

Economic Stability	
Impact of HIV on Finances	*“I don’t think it impacted my finances. I think it helped me out because I got disabled. I can walk still, but I got disabled to find out what it is going to the clinic, different doctors. Told me what happened, what is this… so financial, they gave me social security, all those things.”* *“I've been homeless before. But you can eat in Boston if you just-- you just gotta know where to go. You never starve here.” “No, I don’t have a phone, because I couldn’t afford that for a while, you know how stuff-- they actually cut my hours on the job. They cut me for 40 hours to 20 hours. So, that started making stuff kind of like, real hard. But they're gradually going back up now. A few hours more.”* *“Living from paycheck to paycheck. Yeah, it’s like, it’s kind of hard, because you got to eat, and trying to budget and everything.”* *“Yeah. Well, with my financial issues, I don’t really have a stable job. I do on and off. Sometimes I go, sometimes I don’t. So sometimes I have friends that help me out but like -- and because of my immigration status, that’s [one of the] problem as well.”*
Impact of Competing Responsibilities on HIV Care	*“My mother, she is disabled. She's been disabled for most of my life. I don’t live with her, my brother does, but occasionally, she does need me to come, but it makes it easy most of the time, because she'll have an appointment at the same time. But if not, then it’s like we're both trying to figure out how to do X, Y and Z.”*

**Table 5.** Impact of Neighborhood and Built Environment on Linkage to HIV Care.

Neighborhood & Built Environment	
Neighborhood & Environment	*“I live in [city name]. I don’t really go outside due to work. I work on the computer all day, so when I do get a chance to go outside, it’s pretty good. I don’t have any problems. Neighbors are friendly.”* *“I'm trying to get my own place, it took me that much that long. I wish it was not able to take that long, especially. But then again, you got to -- people are not going to be giving you a place to live if you're freaking selling drug and bringing a bunch of people to your house to do drugs and shit like that. It’s a [expletive] impossible [inaudible] and that happens a lot, because I see it, I know. But, now I'm stable on a place.”* *“I've been living here in my place for 14 years…for my children I feel safe there, they are okay in the neighborhood…I mean, I'm not going put my guards down anyway, because it’s so much going up. Far as we live in here, it’s been decent. It’s been okay. It’s been, I haven’t -- we haven’t had no problems. So I'm grateful for that.”* *“I live in Plainville. I love this area. It’s nice and quiet. I have a swimming pool.* *I have a tennis court. I have a computer room. I have a theater. I have everything where I live, I don’t need nothing, everything's here. All the stores, I got Burger King, McDonald’s across the street, Family Dollar. You name it, I have it down the street, two minutes away from me.”*
Current Housing Situation	*“The neighborhood is lovely, it’s a nice place it’s quiet… I don’t want her [roommate] to come in my room and see the medication, she took it calmly.”* *“I know where to find housing if I need a house. I lived in a shelter for some time, for like two or three years, but I'm now a homeowner. I know where to find resources. I know where to find heat if I need heat. I know where to find A, B, C, D”* *“I bought this house in May of 2018. So I think that’s about five years.”* *“I couldn’t afford to pay my rent. And we moved to my mother's. Right now, we are sharing one room. I need help to get an apartment, and there are two boys so I cannot be, you know how in this country you cannot be in a room with two boys. But, I'm trying to get my stuff for housing. That I need help with that one, for housing.”*
Access to Transportation to HIV Appointments	*“Oh, it’s consistent but economically it is a problem. So I got to get some assistance…I get it through… [hospital], sometimes they help me out with the Charlie pass”* *“I drive.”* *“I take the T, I walk. I ride my bike.”* *“Well, that’s why I bike too a lot because the T can be very… expensive.”* *“The PC one. They take me to my clinic every morning, bring me back home. Then if I need to go shopping or something, they'll accommodate me.”* *“Yeah. I have no problem getting in to see them, because I take an Uber.”*

**Table 6.** Impact of Social and Community Context on Linkage to HIV Care.

Social & Community Context	
Change(s) in Social Life Since HIV Diagnosis	*“I have a lot more social responsibilities…Because I have some things I have to do… They need people like me to talk because I have the experience. So I actually have a lot more social responsibilities.”* *“I had friends that I told, and I thought was my close friends and that would be supportive of it and be on my side to help me, and instead they went and told a whole bunch of people about it [HIV Diagnosis].” "Because I'm like, okay, if I lose them, that means I'm going to be alone. That thought just still comes back to my head. I'm going to be alone for my whole life. I'm never going to have someone who will love me and want to be with me, because I was told and especially around the stigma of it all makes it harder for you to be like, oh yeah, I'm going to--1 have somebody, they love me, they care about me, so on so forth”* *But 90s people got angry with you, if you were positive. Some girls didn’t want to be with you, even you used nothing. Now it’s very different, very very different… People didn’t know before. They didn’t know anything. They didn’t know nothing about it. They talked about it but they didn’t know what they were talking about.”* *“Yeah, my whole family knows about it.”* *“So from experience I don’t go telling everybody I have this, because it’s none of their business. But among my peers it’s fine, because we share, we go for retreats together, you see, and let’s say at my job, I don’t have to tell nobody, it’s none of their business. Because that’s where people start discriminating you and treating you in an evil way.”* *“… I was freshly new in the country. I didn’t really have a lot of support or anything like that. So it was kind of hard for me but then everything just working.”*
Personal & *Community* Views on HIV	*(I) was handed water, and in (my) country (I) would be handed water farther away.* *“I used to have a girlfriend that every time I went to her house, I went to the bathroom she thought I didn’t know where she would go in the bathroom and wash the toilet down with bleach.”* *“Now I feel like people don’t care. People don’t care much, they have commercials in TV, they see people… all the things, married, men, women. All those things change everything.” (007)* *“Well, I see it, it’s coming a long way. There's a lot of good medication out there. People are living longer like me, for example. Living longer and they're functioning better. Yeah, there's good treatments out there now. You gonna take the advantage of it and I believe there's a cure out there because if they can control it, they can have a cure.”* *“So, doctors and nurses were even afraid to treat us, because they thought that by touching us, by treating us, we were going to pass the virus to them. So it was really very difficult. So there are so many trainings and seminars that were introduced by the government, because the government got funding from the US from all over the world. So they put in a lot of money to educate everybody and especially the healthcare workers. And then slowly people started to accept us.”*
Friends, Family, and/or Community Knowledge of HIV Diagnosis	*“So, my significant other, we just got together about three years ago. And I told him in the very beginning… And he said that’s fine. He knows multiple people who have it, it doesn’t bother him… So it felt good to feel accepted for what I had and all of that.”* *“Yeah, my whole family knows about it.”* *“At first it was kind of hard because I didn’t expect it. But, you know, my brother also is HIV. So he's been like that longer than me. I have a twin brother. He's been HIV, more like since 1998 or something. But he's been taking care of himself very well and taking his medication. So we always keep each other, you know, maintaining ourselves.”* *“So I was introduced to a support group and when I went there, I was welcomed with loving arms from other people who looked like me who shared the same status. So I started going…and they were teaching us about positive living, coping with your status and things like those a lot of education.”* *“Like, relationship-wise, mentally sometimes. Like, when you sit down and start thinking if this hasn’t happened, I would be doing this. I would be going there. I would be living a better life or something like that. So it affects me a lot.”*

**Table 7.** Participan’ts Perceived Needs to Improve Linkage to HIV Care.

Perceived Needs & Recommendations for Improvement	
Recommendations to Improve HIV Care	*“There’s an organization I just thought of, it came in a dream that I want to start. It’s called "A shoulder to cry on.” It’s when people get bad news health-wise, we would be those people, to give you a shoulder to cry on…”* *“I feel like not just support groups, but support groups for different ages, because I've been invited to multiple support groups, but these support groups have like children in them. I'm an adult, so it feels kind of weird to be at a support group with young children.”* *“Yeah, to call you or see you whatever, personally, I don’t know. I prefer a visit instead of calling because I can’t judge you, not judge you but you know what I mean? The point you can say anything you want and you might be, but not in my face. So, but yeah, more communication. I think that’s about it for me, communication is everything for me.”* *“Maybe sometimes I feel like talking with somebody. I should get therapy maybe one time or two times a month to help me out.”* *“The way we are treated when we go in that door over there. Oh, the first thing all we want is drugs and that’s not it. I think it should be less hostile. A less hostile environment to really get people to come in. It has to be, well, people won’t still be dying.”* *“Just cheaper medicine. I mean, I would want the best medicine, but I don’t know how to pay $50 for one bottle. That’s about it.”* *“So, people, who are in this current generation, they have everything they need. They only need to look for health care where they can go get tested, and get medication, get counseling, there's a lot of therapy and things like those and there's a lot of networking.”*
Recommendations to Improve Access to HIV Appointments and Medication	*“It’s like sometimes my emotions play with me. That’s the only reason sometimes I don’t go to my appointments.”* *“So when it comes to health insurance and everything and having so much issues, getting medication or not having insurance or having insurance, it makes it harder to want to even go to the appointment, because for some females that I did speak with, they're on a handful of medications. I'm lucky enough to be one of the people who have one pill a day"”* *“I'm still In a state of denial… That’s why I have problems with medications, so back then they were pushing all these pills… I didn’t like that at all. So I went without medication for a while, they made some kind of thing just to take one pill, one pill every day. But it was, it was like a reminder for me. You know what I mean? That’s what I hate about that.”*
Recommendations to Improve HIV Programs & Overall Experience	*“No, we used to be in the basement. The infectious disease clinic was in the basement. Now, we're on the top door of the…building. So we have a great view…They're done trying to hide us because we're such a large number of people now. We were hidden before.”* *“Yeah, group sessions help a lot, a lot…from my experience that helps a lot, because you go to a bunch of other people there and you're saying a predicament, and they might be going through some shit they could related -- they can relate and they're doing something about it that I might not think of.”* *“ mean, it’s good to know different people that…I'm into the group and knowing more how they carry themselves through, you know, dating malfunctioning when they, you know, are HIV positive and they've been very helpful and talk about it, you know, feelings and stuff like that. It’s been dne.”* *“So they mainly focused on… Black women living with HIV and AIDS, and we had these leaders who have been in the process for 20, 40 years, you know what I'm saying? And they talking to the crowds it’s like they were, there's [a] word I want to use but it’s not coming. Rallying, they were rallying the crowds to fight for themselves, to fight for their rights. And we had people from the government also in the conference. Black women who work in the government, who are helping bring out the issues. People who are in policy and they're out there specifically to provide funds that would empower the Black women living with HIV and AIDS in the US.”*
